# Clinical and Immunologic Profiles in Incomplete Lupus Erythematosus and Improvement with Hydroxychloroquine Treatment

**DOI:** 10.1155/2016/8791629

**Published:** 2016-12-28

**Authors:** Nancy J. Olsen, Carl McAloose, Jamie Carter, Bobby Kwanghoon Han, Indu Raman, Quan-Zhen Li, Duanping Liao

**Affiliations:** ^1^Division of Rheumatology, Department of Medicine, Penn State MS Hershey Medical Center, Hershey, PA, USA; ^2^Division of Rheumatology, Department of Medicine, University of Washington, Seattle, WA, USA; ^3^Department of Immunology, University of Texas Southwestern Medical Center, Dallas, TX, USA; ^4^Department of Public Health Sciences, Penn State University, College of Medicine, Hershey, PA, USA

## Abstract

*Objective*. The study goals were to evaluate performance of SLE classification criteria, to define patients with incomplete lupus erythematosus (ILE), and to probe for features in these patients that might be useful as indicators of disease status and hydroxychloroquine response.* Methods*. Patients with ILE (*N* = 70) and SLE (*N* = 32) defined by the 1997 American College of Rheumatology criteria were reclassified using the 2012 Systemic Lupus International Collaborating Clinics criteria. Disease activity, patient reported outcomes, and levels of Type I interferon- (IFN-) inducible genes, autoantibodies, and cytokines were measured. Subgroups treated with hydroxychloroquine (HCQ) were compared to patients not on this drug.* Results*. The classification sets were correlated (*R*^2^ = 0.87). ILE patients were older (*P* = 0.0043) with lower disease activity scores (*P* < 0.001) and greater dissatisfaction with health status (*P* = 0.034) than SLE patients. ILE was associated with lower levels of macrophage-derived cytokines and levels of expressed Type I IFN-inducible genes. Treatment of ILE with HCQ was associated with better self-reported health status scores and lower expression levels of Type I IFN-inducible genes than ILE patients not on HCQ.* Conclusion*. The 2012 SLICC SLE classification criteria will be useful to define ILE in trials. Patients with ILE have better health status and immune profiles when treated with HCQ.

## 1. Background

A significant number of individuals seeking care in rheumatology clinics have some findings suggestive of SLE but do not have 4 of the defined classification criteria. Some of these individuals are identified through anti-nuclear antibody (ANA) screening that was carried out for nonspecific symptoms. Others have cutaneous complaints and are seen by dermatologists. A patient with significant ANA positivity and a photosensitive malar rash, for example, does not fit classification criteria for SLE, but the findings may be of concern and warrant further evaluations or treatment. This is especially true for young females, who are in the highest risk group for development of SLE. Such a patient may be considered to have incomplete lupus or ILE. This terminology has been used for many years and has implications for lupus risk [1, 2]. Patients with ILE are often treated with low doses of prednisone or with hydroxychloroquine and monitored for development of further lupus manifestations. At least one study from a university-based dermatology clinic suggests that some patients who present with an incomplete and largely cutaneous symptom complex do progress to SLE but have a low risk of developing organ-damaging manifestations [3]. But concerns also have been raised about whether overuse of the ILE terminology is inappropriate in view of the fact that many will never develop SLE or another significant illness [4, 5]. Determining which ILE patients are at greatest risk has some urgency, as it will open possibilities for interventional trials to modify disease and reduce progression to SLE.

The goals of the current study were to characterize patients with ILE who were seen in an academic rheumatology clinic and to evaluate approaches to classification and outcome assessment that could be applicable to clinical trial interventions. Effects of one potential intervention, hydroxychloroquine (HCQ), on clinical and immune parameters were determined. The results suggest that this treatment has benefits in ILE and potential to modulate long-term outcome in these patients.

## 2. Methods

### 2.1. Subjects

Patients with lupus syndromes were identified during routine care in the clinics of the rheumatology division. SLE was defined using the 1997 ACR criteria [6]. ILE was defined as any individual who had accumulated 1 to 3 of the ACR criteria. Each individual was then reclassified using the 2012 SLICC set of criteria [7]. Individuals with ANA as the only criterion also had other clinical or immunologic findings suggestive of autoimmunity and disease risk such as positivity for Ro/SSA or other autoantibodies, presence of Raynaud's, or strong history of autoimmune disease in one or more first degree relatives. Most of these individuals were being followed regularly in the outpatient clinics because of these ongoing concerns. Some results were also compared to 13 healthy control (HC) subjects. The research protocol followed Declaration of Helsinki principles and was approved by the Penn State Hershey Institutional Review Board. All patients provided written informed consent prior to any study-related activities.

### 2.2. Blood Samples

Peripheral blood samples were drawn for collection of serum, stored as frozen aliquots at –80°C, and drawn separately into PAXgene tubes that were also frozen and used for later isolation of RNA.

### 2.3. ANA and Autoantibody Profiles

The ANA was performed as part of usual clinical care and carried out in the hospital laboratory using the indirect immunofluorescence assay (IFA) on Hep-2 cells that was used to score the classification criteria; titers of 1 : 80 or greater were considered positive. Other autoantibodies used for classification (double-stranded DNA, Sm, anti-phospholipid) were measured in the clinical laboratory as part of standard care and were obtained by chart review. Each ILE and SLE patient also had measurement of ANA carried out in the research laboratory on serum samples using an immunoassay (Inova, San Diego CA) and results were expressed as Elisa Units (EU) using standards provided with the kit, as described previously [8]. Values that are greater than 20 U in this assay are considered positive.

### 2.4. Self-Assessment Questionnaire

Ten questions from the Multi-Dimensional Health Assessment Questionnaire (MDHAQ) regarding ability to perform daily activities were scored on a scale of 0–3 and expressed as an average [9]. Fatigue and pain in the past week as well as global health status were rated on 10 cm continuous visual analog scales. Two additional questions were as follows.

(1) In general would you say that your health is (scores shown in parentheses):

Excellent (score = 1)
Very good (2)
Good (3)
Fair (4)
Poor (5)

(2) Compared to one year ago, how would you rate your health in general now?

Much better than one year ago (score = 1)
Somewhat better than one year ago (2)
About the same as one year ago (3)
Somewhat worse than one year ago (4)
Much worse than one year ago (5)

### 2.5. Immunoassays

Commercial Elisa kits were used for serum measurements of C reactive protein (CRP) (Abnova, Taipei, Taiwan), anti-C1q (Novus Biologicals, Littleton CO), soluble CD27 (sCD27; eBioscience, San Diego, CA), antithyroglobulin (Novus), and IgM-rheumatoid factor (IgM-RF; Alpha Diagnostics, San Antonio, TX).

### 2.6. Cytokines

Levels of cytokines were measured in serum samples using the Bio-Rad Human 27-Plex array (Bio-Rad, Hercules, CA, USA) which uses a magnetic bead-based technique [10]. Results are expressed as the mean of two replicates in pg/mL using standard curves provided by the manufacturer for each cytokine.

### 2.7. Gene Expression

Total RNA was purified from PAXgene tubes and quantitated with a NanoDrop 2000 (Thermo Scientific, Wilmington, DE, USA). Preparation of cDNA was done using the High Capacity RNA-to-cDNA Kit (Applied Biosystems/Life Technologies, Carlsbad, CA, USA) with 100 to 200 ng RNA per synthesis reaction. RT-PCR analysis was performed for selected genes using TaqMan Gene Expression Assays (Life Technologies) with GAPDH as the housekeeping control gene with an ABI-7300 Real-Time PCR instrument. Expression values are normalized to GAPDH levels using the following formula: 2^(GAPDH  Ct − Test  gene  CT)^ as previously described [11].

### 2.8. Statistical Analyses

Data are shown as mean values and standard errors of the means. Correlations between pairs of continuous variables were done using Pearson's *r*. Dichotomized variables were compared between subject groups using Fisher's Exact Chi-Square Test. Bonferroni correction was applied in some of the analyses. All *P* values were from 2-sided tests and values of <0.05 were considered significant. Analyses and graphics were carried out using SAS version 9.3 software (SAS Institute Inc., Cary, NC, USA) and Prism version 6.0 (Graph Pad, San Diego, CA, USA).

## 3. Results

### 3.1. Classification, Demographics, and Health Status of ILE versus SLE

More than 94% of patients were female (Table 1), greater than 80% were Caucasian, and more than 90% were non-Hispanic, with no significant differences between the ILE and SLE groups (data not shown). Mean age was greater in ILE (45.3 ± 1.6 years (mean ± SEM)) than in SLE (37.3 ± 2.1 years; *P* = 0.005). The range of ages was also greater for ILE (18–74 years) than for SLE (20–60 years); 41% of ILE patients were older than 50 years while only 15% of SLE patients were in that age range (*P* = 0.0029). The HC group (*N* = 13) was predominantly female (92%) and had an average age of 32 years; this was not significantly different from the mean age of SLE patients but was less than the mean value for the ILE group (*P* ≤ 0.01).

The number of ACR criteria satisfied was significantly different between ILE and SLE (Table 1), consistent with the way the groups were defined. Disease activity measured by SLEDAI was low in both groups; only four patients had scores > 6. However the mean value was significantly higher in SLE than ILE (*P* = 0.003; Table 1). ANA levels measured by Elisa were not significantly different in ILE than in SLE (63.41 ± 5.8 EU versus 81.3 ± 10.44 EU; *P* = 0.11). Measures of fatigue, pain, and difficulty scored on the MHAQ were not significantly different in the two groups (Table 1). Self-assessed health status was significantly better in ILE (2.84 ± 0.11) than in SLE (3.55 ± 0.17; *P* = 0.0005). However when asked to rate their current health status compared to a year previously, the ILE patients had a significantly higher average score (3.24 ± 0.13), corresponding to slightly worse than the descriptor “about the same,” than those with SLE, in whom the average score was significantly better (2.71 ± 0.23; *P* = 0.034). The most commonly used lupus specific medication was hydroxychloroquine, taken by 21% of ILE and 65% of SLE patients (*P* < 0.0001). For the complete group of patients, including both ILE and SLE, those who were currently taking HCQ rated their health status as better than those who were not on this medication (2.53 ± 0.20 versus 3.30 ± 0.11; *P* = 0.0005).

**Figure 1:**
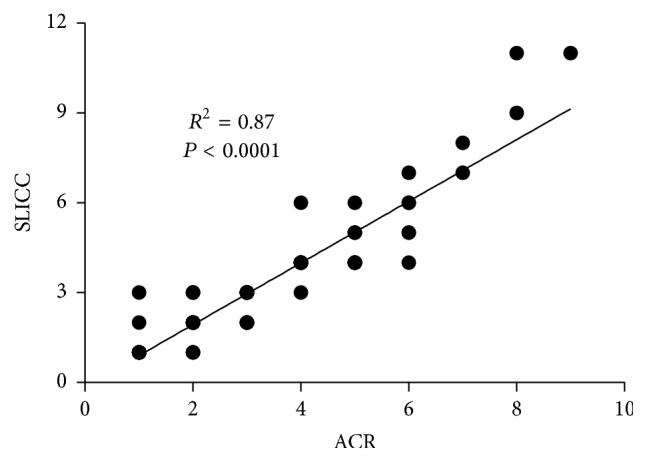
Correlation between two classification SLE criteria, the 1997 ACR and 2012 SLICC sets, in 102 patients with either ILE or SLE. Values on each axis correspond to numbers of criteria in each of the sets. Significance determined using Pearson's *R*.

All enrolled individuals were classified first with the 1997 ACR criteria and then with the 2012 SLICC criteria. The two datasets were highly correlated (*R*^2^ = 0.87; *P* < 0.0001; Figure 1). The same levels of correlation and significance were obtained when the 19 individuals who had only ANA positivity (1 criterion) in both sets of criteria were removed. Two of these 19 patients had more than one of the SLICC criteria. Only one individual changed from SLE, with 4 of the ACR criteria, to ILE with only 3 of the SLICC criteria. Significant differences in the prevalence of specific features in ILE compared to SLE were examined for the two sets of classification criteria. When corrected for multiple comparisons, 6 of the 11 ACR criteria were significantly higher in SLE than in ILE (Table 2). These were malar rash, photosensitivity, oral ulcers, serositis, hematologic disorder, and immunologic disorder (*P*_corr_ < 0.01 for each). The SLICC criteria yielded similar findings, with 6 of the 17 criteria being significantly higher in SLE than in ILE: acute cutaneous, oral ulcers, serositis, leukopenia, and antibodies to Sm and dsDNA (Table 3; *P*_corr_ ≤ 0.05 for each). None of the ILE patients had renal or neurologic criteria using either classification set. Viewed qualitatively, the criteria with significant differences between ILE and SLE are in similar categories, involving skin, mucosa, serosa, hematologic abnormalities, and autoantibodies. These results confirm that the SLICC classification matches closely the previous ACR version when ILE patients are included in the analysis and suggest that classification using the 2012 SLICC criteria will not reduce prevalence of the ILE designation, as has been suggested [12].

### 3.2. Gene Expression and Immune Variables in ILE and SLE

**Figure 2:**
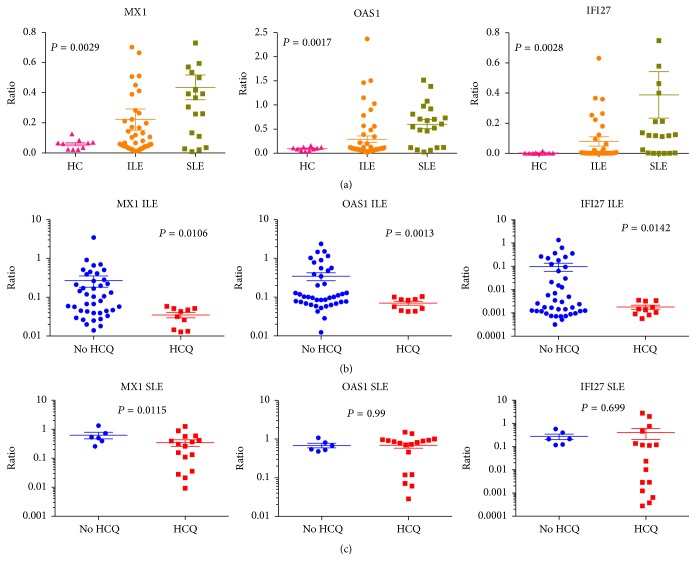
Expression levels of three specificities in the Type I IFN signature, MX1, OAS1, and IFI27, measured by RT-PCR, are shown in patients with ILE and SLE: (a) HC, ILE, and SLE groups; (b) ILE patients grouped by HCQ use; (c) SLE patients grouped by HCQ use. Values are expressed as a ratio relative to GAPDH, as described in the methods section. Individual data points are shown, with mean and SEM values also indicated. For 3-group comparisons in (a), 1-way ANOVA was used to calculate significance. HCQ and non-HCQ groups were compared in (b) and (c) using Student's *t*-test.

Expression of the Type I IFN gene signature that is elevated in SLE was probed by measuring expression levels of three specificities, MX1, OAS1, and IFI27. Each of these three genes was expressed at a higher level in SLE group than in ILE (Figure 2(a)). Differences were significant for OAS1 (*P* = 0.0108) and IFI27 (*P* = 0.0061) but not for MX1 (*P* = 0.0846). Both IFI27 and OAS-1 were significantly correlated with the number of ACR criteria (*P* = 0.0021 and *P* = 0.047, resp.). The specificity most closely related to SLE was IFI27, confirming our previous findings [13].

A panel of immune parameters was measured in ILE and SLE (Table 4(a)). Two of these were autoantibodies associated with diagnoses other than SLE that ILE patients might differentiate into IgM-RF for rheumatoid arthritis and antithyroglobulin (TG) for autoimmune thyroid disease. Other markers were CRP for inflammation and cardiovascular risk, sCD27 associated with lymphocyte activation in autoimmune diseases, and anti-C1q associated with nephritis in SLE [14]. Only anti-C1q was significantly lower in ILE than in SLE (*P* = 0.045), consistent with the association of this specificity with nephritis, which is absent in the ILE patients. Levels of anti-C1q also showed significant correlation with the number of ACR criteria and with SLEDAI score (*P* < 0.0001 for each).

### 3.3. Cytokines and Chemokines

Measurement of soluble mediators including cytokines and chemokines on a multiplex array was carried out to evaluate ILE/SLE differences as well as effects of HCQ in ILE. Five cytokines, IL-9, IP10, MCP-1, MIP1 alpha, and TNF alpha, were significantly higher in SLE than in ILE (Table 5(a)). In addition, IL-13 showed a statistically significant but relatively low level of correlation with the number of SLE criteria (*R*^2^ = 0.15; *P* = 0.020).

### 3.4. Effects of HCQ Treatment in ILE

Use of HCQ in early disease stages has been proposed as a preventive strategy in SLE [15]. To develop insights into this approach, the SLE and ILE patients were further examined in subgroups defined by current HCQ usage. The ILE subgroup treated with HCQ rated their overall disease compared to a year ago significantly better than ILE patients who were not on HCQ (*P* = 0.0005) and also had lower modified health assessment questionnaire scores (*P* = 0.0146).

The HCQ-ILE group also had significantly lower expression levels of all three of the measured Type I IFN-inducible genes (*P* ≤ 0.01; Figure 2(b)), suggesting that treatment with HCQ has quenching effects on the IFN signature. The SLE patients in HCQ and non-HCQ subgroups did not show significant differences in IFN-inducible gene expression, but it was clear that the lowest levels for all 3 genes were in the SLE-HCQ patients (Figure 2(c)).

For the immune assays, the mean anti-C1q level was significantly lower in the ILE-HCQ group compared to the ILE patients who were not on this drug (0.99 ± 0.57 versus 4.18 ± 1.12; *P* = 0.013; Table 4(b)). In the cytokine array, analysis of HCQ-defined ILE subgroups showed only IL-9 to be significantly different in HCQ versus non-HCQ treated patients (18.67 ± 1.88 versus 29.56 ± 3.14; *P* = 0.00046; Table 5(b)).

## 4. Discussion

Approaches to classifying ILE and to assessing risks of SLE in this population are needed, to perform approaches to clinical care and to understand the pathogenesis of lupus syndromes. Tools will be necessary to organize intervention trials in the ILE population that may be designed to develop insights into SLE prevention. The results of the present study indicate that the 2012 SLICC classification criteria can be used to define the ILE population for clinical trials. In terms of looking for early disease candidates for such trials, these criteria are more sensitive than the ACR criteria, in both adult and pediatric populations [7, 12, 16], offering potential benefits in screening for patients at risk. Furthermore, the greater number of criteria, 17 versus 11, offers more range of possible change as an outcome measure. One previous report had suggested that the new criteria might result in a reduction in the frequency of an ILE classification result [12], but that does not appear likely. Approximately half of the ILE patients in this study were greater than 50 years of age, representing a lower risk group for development of lupus. For a trial to test prevention strategies, it will be important to focus on younger patients.

The general distributions of specific categories in the two sets of criteria that appear in ILE and SLE were very similar. One change in the 2012 criteria is the loss of photosensitivity as a separately scored point. Photosensitivity is a frequent feature of patients with unclassifiable lupus-like syndromes, present in more than 20% of these patients [17], but the loss of this as a separate criterion did not appear to have a great impact on the ILE scores in the present study. Another change in the 2012 criteria is the addition of low complement as scored item. In the present study, 6 of the ILE patients (8.9%) had low complement levels, and none of these individuals had positivity for any of the autoantibodies that would have been scored under the ACR immunologic category (anti-dsDNA, anti-Sm, and antiphospholipid). In some of these ILE patients, loss of scoring for photosensitivity was counterbalanced by the addition of hypocomplementemia, resulting in no change in the overall score. As in most of the other reports, renal and neurologic criteria were rare or absent in ILE [18] and the more specific antibodies such as dsDNA were also significantly less prevalent than in SLE.

Relatively simple demographics and clinically available variables can be very useful in assessing SLE risk [19]. Female gender, age less than 40 years, and high levels of ANA positivity are readily identifiable variables which can identify a subgroup of ILE patients at risk for development of SLE [20]. However, biomarkers that would assist with early diagnosis of SLE or that would aid in assessment of risk prediction are needed to help guide referral practices of primary care physicians, counsel individual patients in clinical practice, and design trials that would target those patients who are at greatest risk for progression [21]. In clinical care, repeated and inconclusive evaluations may go on for many years. In one cohort, 56% of patients identified as “potential” SLE remained in that category after a mean of 6.3 years of followup [17]. This long period of diagnostic uncertainty may explain the somewhat surprising finding of the self-assessment responses in the ILE group reported here, showing that these patients thought that they were actually worse than they had been a year previously, even though a diagnosis of a more serious illness, SLE, had not been made.

Biomarkers of SLE risk include the Type I IFN signature, which is elevated in about half of ILE patients [13]. The present study confirms previous results showing that IFI27 is one specificity of this signature which is less likely to be elevated in ILE than SLE, suggesting potential utility as an indicator of lupus risk. Other useful markers of risk or disease progression in ILE patients will likely include soluble mediators and autoantibodies [20, 22, 23]. Candidate autoantibody specificities investigated in the present study include C1q, levels of which were associated with number of criteria and SLEDAI score. This observation is consistent with the association of anti-C1q with nephritis, which is not present in ILE [14], and suggests that the presence of an elevated anti-C1q in a patient with ILE might raise concerns for SLE or more specifically, nephritis. Few ILE patients in this study had significant levels of C1q, so sensitivity for detection of impending nephritis may be low, and it remains undetermined whether anti-C1q is, like anti-dsDNA, a demonstrated predictor of SLE risk [17].

Five cytokines were significantly lower in ILE than in SLE patients. Three of these, IP10, MCP-1, and MIP1*α* are chemokines that are associated with Type I IFN and have been reported to be elevated in individuals who later develop SLE [22, 24]. Another, TNF*α*, has been shown to have a central role in mediating cutaneous inflammation in lupus syndromes [25]. IL-9 is a Th2 or Th9-derived cytokine that has also been implicated in the pathogenesis of SLE [26], possibly by expanding the Th17 cell population [27].

The ILE population showed significant differences in subsets defined by the use of HCQ, with patients on this responding more positively about their health status than ILE patients not on HCQ. The Type I IFN-inducible genes were also significantly lower in the HCQ-ILE subset, consistent with in vitro studies demonstrating that HCQ blocks production of Type I IFN by dendritic cells from patients with SLE [28]. A subset of SLE patients treated with HCQ also showed low levels of the IFN genes, although differences with the non-HCQ group were not statistically significant in the small sample tested. The lack of effect of HCQ on expression of Type I IFN-inducible genes in some SLE patients is consistent with the concept proposed elsewhere that once the immune response is amplified, which would be anticipated in SLE more than in ILE, it becomes difficult to arrest the abnormalities [29]. The finding of clinical benefit and decreased expression of the Type I IFN signature is also consistent with other reports showing a correlation between disease activity in SLE patients and circulating IFN-a levels [30]. Significant efforts are being made to develop anti-IFN therapeutics for SLE [31]. The potential for HCQ to modulate this signature may contribute substantially to its therapeutic effects, especially in early stages of disease.

The limitations of this study include the small sample size and the cross-sectional design. Longitudinal studies of ILE patients in larger cohorts will be required to determine whether any of the mediators or expressed genes identified in the present study can be used to reliably identify those patients who are at risk for progressive disease. The definition of ILE used here was intentionally broad, in order to be exploratory and hypothesis-generating. Trials to evaluate preventive strategies will need to focus on higher risk younger patients, with at least 2 SLICC criteria.

## 5. Conclusions

The 2012 SLICC SLE classification criteria will be useful in defining patients with ILE syndromes in longitudinal observational or interventional trials. Patients with ILE have self-assessment metrics suggesting the perception that they are not doing well over time, reflecting burdens of illness and anxiety that may be underrecognized by providers. The use of HCQ in ILE may prevent progression to SLE by modulation of Type I IFN pathways. Longitudinal, controlled studies of this intervention will be of interest.

## Figures and Tables

**Table 1:** Characteristics of ILE and SLE study groups.

Feature	ILE (*N* = 70)	SLE (*N* = 32)	*P*
Age (years)	45.3 ± 1.6	37.3 ± 2.1	0.005
Female/male	66/4	31/1	1.0
ACR criteria	2.0 ± 0.1	5.3 ± 0.2	4 × 10^−29^
SLEDAI score	1.31 ± 0.21	4.00 ± 0.60	<0.001
ANA (EU)	63.41 ± 5.77	81.32 ± 10.44	0.30
Fatigue (mm)	5.46 ± 0.38	5.59 ± 0.61	0.85
Pain (mm)	3.65 ± 0.35	4.23 ± 0.46	0.33
mHAQ (0–3)	1.40 ± 0.05	1.52 ± 0.09	0.22
Health status (1–5)	2.84 ± 0.11	3.55 ± 0.17	0.0005
Rate current health (1–5)	3.24 ± 0.13	2.71 ± 0.23	0.034
Hydroxychloroquine	15 (21%)	21 (65%)	<0.0001
Cyclophosphamide	0 (0%)	1 (3%)	0.31

*P* values are calculated by *t*-test or Fisher's Exact Test for dichotomized variables.

**Table 2:** ACR classification criteria in ILE and SLE patients.

Criterion	ILE (*N* = 70)	SLE (*N* = 32)	*P*	*P*_corr_
Malar rash	11 (16)	19 (59)	<0.0001	*∗∗*
Discoid rash	1 (1)	5 (16)	0.0111	NS
Photosensitivity	16 (23)	24 (75)	<0.0001	*∗∗*
Oral ulcers	9 (13)	21 (66)	<0.0001	*∗∗*
Arthritis	14 (20)	14 (44)	0.0172	NS
Serositis	6 (9)	15 (47)	<0.0001	*∗∗*
Renal	0 (0)	4 (13)	0.0085	N/A
Neuro	0 (0)	3 (9)	0.0289	N/A
Heme	5 (7)	16 (50)	<0.0001	*∗∗*
Immunologic	8 (11)	17 (53)	<0.0001	*∗∗*
ANA	68 (97)	32 (100)	1.00	N/A

Values represent number of individuals (percent).

*P* values are calculated by Fisher's Exact Test.

*P*corr are values after Bonferroni correction of multiple (8) comparisons:

^*∗∗*^ *P* value less than 0.00125.

NS: not statistically significant; N/A–cell = zero; no statistical test was done.

**Table 3:** SLICC 2012 classification criteria in ILE and SLE patients.

Criterion	ILE (*N* = 71)	SLE (*N* = 31)	*P*	*P*_corr_
*Clinical*				
Acute cutaneous	16 (22.5)	20 (64.5)	<0.0001	*∗∗*
Chronic cutaneous	1 (1.4)	5 (16.1)	0.0095	NS
Oral ulcers	9 (12.7)	21 (67.7)	<0.0001	*∗∗*
Alopecia	1 (1.4)	0 (0)	1.00	N/A
Synovitis	14 (19.7)	16 (51.6)	0.0019	*∗∗*
Serositis	7 (9.9)	15 (48.4)	0.0001	*∗∗*
Renal	0 (0)	4 (12.9)	0.0074	N/A
Neurologic	0 (0)	3 (9.7)	0.0262	N/A
Hemolytic	1 (1.4)	2 (6.5)	0.2185	NS
Leukopenia	4 (5.6)	11 (35.5)	0.0003	*∗∗*
Thrombocytopenia	2 (2.8)	3 (9.7)	0.1630	NS

*Immunologic*				
ANA	70 (98.6)	31 (100)	1.0	N/A
dsDNA	3 (4.2)	13 (41.9)	<0.0001	*∗∗*
Sm	1 (1.4)	7 (22.6)	0.0009	*∗*
APL	4 (5.6)	5 (16.1)	0.1261	NS
Complement	6 (8.5)	10 (32.3)	0.0057	NS
Coombs	0 (0)	0 (0)	N/A	N/A

See Table 2 footnotes. *P* corr significance level after Bonferroni correction for multiple (13) comparisons: ^*∗*^*P* value less than 0.0038; ^*∗∗*^*P* value less than 0.00077.

**(a):** 

Variable	ILE	SLE	*P*
ANA (EU)	63.41 ± 5.77	81.30 ± 10.44	0.11
CRP (mg/L)	7.77 ± 0.672	6.18 ± 1.11	0.21
C1q (U/ml)	3.53 ± 0.91	15.69 ± 5.77	0.045
IgM-RF (IU/ml)	32.34 ± 5.42	22.93 ± 2.70	0.37
Anti-TG (IU/ml)	45.40 ± 4.50	47.12 ± 7.98	0.048
sCD27 (U/ml)	16.07 ± 3.28	43.06 ± 19.70	0.19

**(b):** 

Variable	ILE		SLE		*P*_1_	*P*_2_
	No HCQ	HCQ	No HCQ	HCQ		
ANA	64.18 ± 6.74	60.36 ± 6.74	94.53 ± 19.16	74.40 ± 12.44	0.74	0.37
CRP	7.91 ± 0.75	7.19 ± 0.75	4.49 ± 1.33	8.78 ± 1.63	0.67	0.06
C1q	4.18 ± 1.12	0.99 ± 0.57	8.81 ± 6.51	14.51 ± 8.68	0.013	0.66
IgM-RF	33.90 ± 6.60	26.43 ± 7.54	22.85 ± 0.76	23.05 ± 7.06	0.59	0.97
Anti-TG	45.46 ± 5.43	45.17 ± 6.15	66.38 ± 29.06	41.17 ± 5.22	0.98	0.17
sCD27	14.91 ± 3.22	20.73 ± 10.43	35.34 ± 30.75	45.20 ± 24.46	0.48	0.81

*P* values are calculated by *t*-test.

*P*_1_ compares ILE patients in non-HCQ and HCQ groups.

*P*_2_ compares SLE patients in non-HCQ and HCQ groups.

**(a):** 

Cytokine	ILE	SLE	*P* ^*∗∗*^
IL-9	27.54 ± 2.64^*∗*^	54.66 ± 23.21	0.05
IP10	903.3 ± 72.62	2090 ± 939	0.028
MCP-1	45.68 ± 4.32	78.87 ± 15.92	0.006
MIP1*α*	10.86 ± 4.01	364.5 ± 355.3	0.032
TNF*α*	52.06 ± 5.58	100.3 ± 36.83	0.048

**(b):** 

Cytokine	ILE		SLE		*P*_1_	*P*_2_
	No HCQ	HCQ	No HCQ	HCQ		
IL-9	29.56 ± 3.14	18.67 ± 1.88	82.67 ± 48.74	29.76 ± 4.75	0.00046	0.31
IP10	946.6 ± 87.75	730.3 ± 78.62	3286 ± 1961	1027 ± 207	0.0744	0.29
MCP-1	43.50 ± 4.88	53.96 ± 9.28	94.03 ± 29.39	67.50 ± 17.84	0.3302	0.46
MIP1*α*	12.26 ± 4.84	4.32 ± 0.60	721.8 ± 709.8	7.29 ± 1.5	0.46	0.38
TNF*α*	52.12 ± 6.23	51.83 ± 13.26	164.5 ± 73.8	43.23 ± 5.13	0.98	0.14

^*∗*^Values shown represent mean fluorescence intensity ± SEM.

^*∗∗*^ *P* values are calculated by *t*-test.

*P*_1_ compares ILE patients in non-HCQ and HCQ groups.

*P*_2_ compares SLE patients in non-HCQ and HCQ groups.
